# The Medical Society of Virginia

**Published:** 1874-11

**Authors:** 


*The Medical Society of Virginia.*

The Medical Society of Virginia convened at Abingdon on the 13th to 16th of October.

The profession of the State was represented by a large number of delegates, including many eminent and distinguished men.

The address of welcome by Ex-Governor Robertson, in behalf of the city, was elegant and appropriate. The same may be said of the address of Dr. E. Campbell, who represented the Academy of Medicine.

Dr. S. C. Gleaves, of Withville, was chosen President for the ensuing year. Dr. C. Thompson, Corresponding Secretary, and L. B. Edwards, Recording Secretary.

The Annual Address was delivered by Dr. M. P. Christian, of Lynchburg.

A sumptuous banquet was given by the Academy of Medicine, whereat many interesting toasts and responses were made. The entertainment was in full accord with the liberal and genial spirit of the citizens, and did no discredit to the proverbial hospitality of the Old Dominion.

The body was highly complimented by the city press for the eloquence, logic and intelligence of its members.

We always note, with pleasure, everything of a public character that transpires in the grand old State of Virginia—particularly her medical societies. We are proud of the rank which she holds in point of medical talent. In science, as in politics and morals, she still maintains, as she has ever done, a high and enviable position among the States of the Union.

